# Supplementary material for: A new fluorescence-based approach for direct visualization of coat formation during sporulation in Bacillus cereus
Source: Sci Rep. 2023 Sep 13;13:15136. doi: 10.1038/s41598-023-42143-9 (PMC10499802; doi:10.1038/s41598-023-42143-9)
Supplement: Supplementary file 1 — Supplementary Figures. [file 41598_2023_42143_MOESM1_ESM.pdf]

A new fluorescence-based approach for direct visualization of coat formation during sporulation in *Bacillus cereus*

Armand Lablaine, Stéphanie Chamot, Mónica Serrano, Cyrille Billaudeau, Isabelle Bornard, Rut Carballido-Lopez, Frédéric Carlin, Adriano O. Henriques and Véronique Broussolle

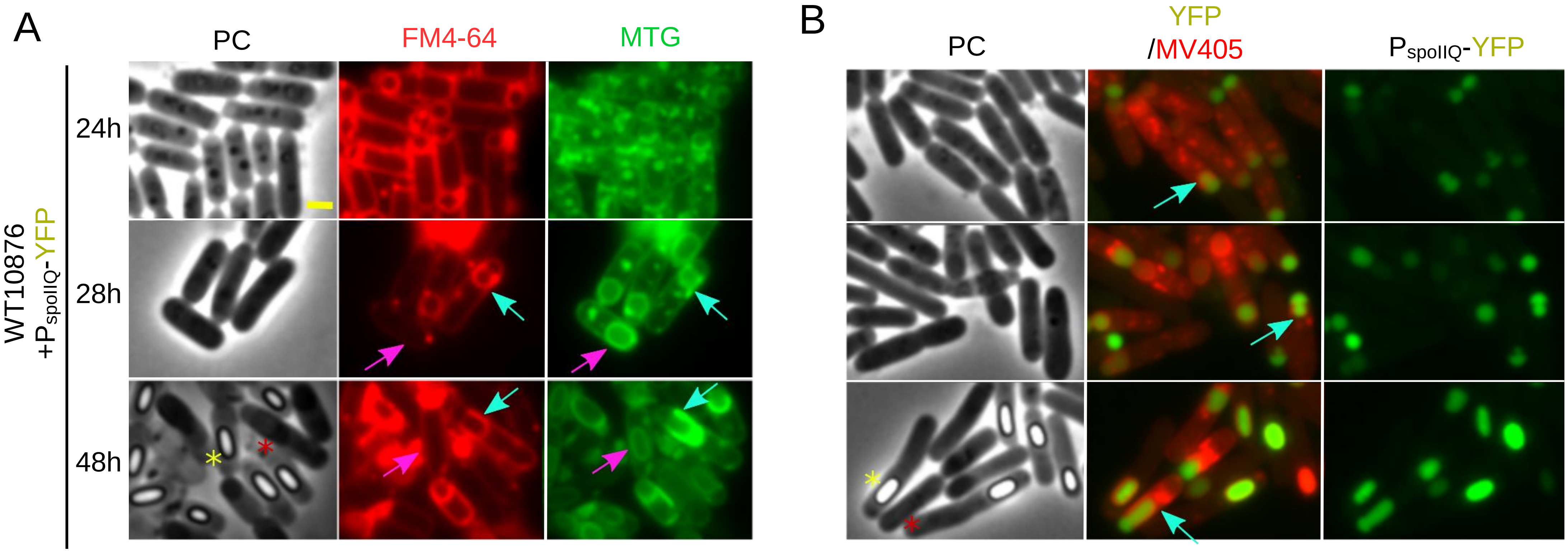

Figure S1. Sporulation at 20°C leads to a forespore bulging which prevents engulfment completion in a subpopulation of sporulating WT10876 cells. *B. cereus* WT10876 cells expressing a P*spoIIQ*-YFP transcriptional fusion as a marker of the forespore and sporulating at 20°C were collected at indicated times and imaged by phase contrast (PC) and fluorescence microscopy, after labeling with the membrane dyes FM4-64 (red) and MTG (green) (**A**) or MV405 (red) (**B**). After engulfment completion, MTG stains the forespore membranes while the lipophilic FM4-64 does not (pink arrows). At hours 28 and 48, the forespore bulge is labelled by both MTG and FM4-64 showing an engulfment defect (cyan arrows). MV405 membrane dye (false colored red) was used to simultaneously visualized YFP protein (false colored green). The YFP is expressed under the *spoIIQ* promoter and confirms the forespore bulging phenotype (cyan arrows). Yellow asterisks indicate refractile forespores and red asterisks point to engulfment-defective cells. Scale bar in A represents 1 µm.

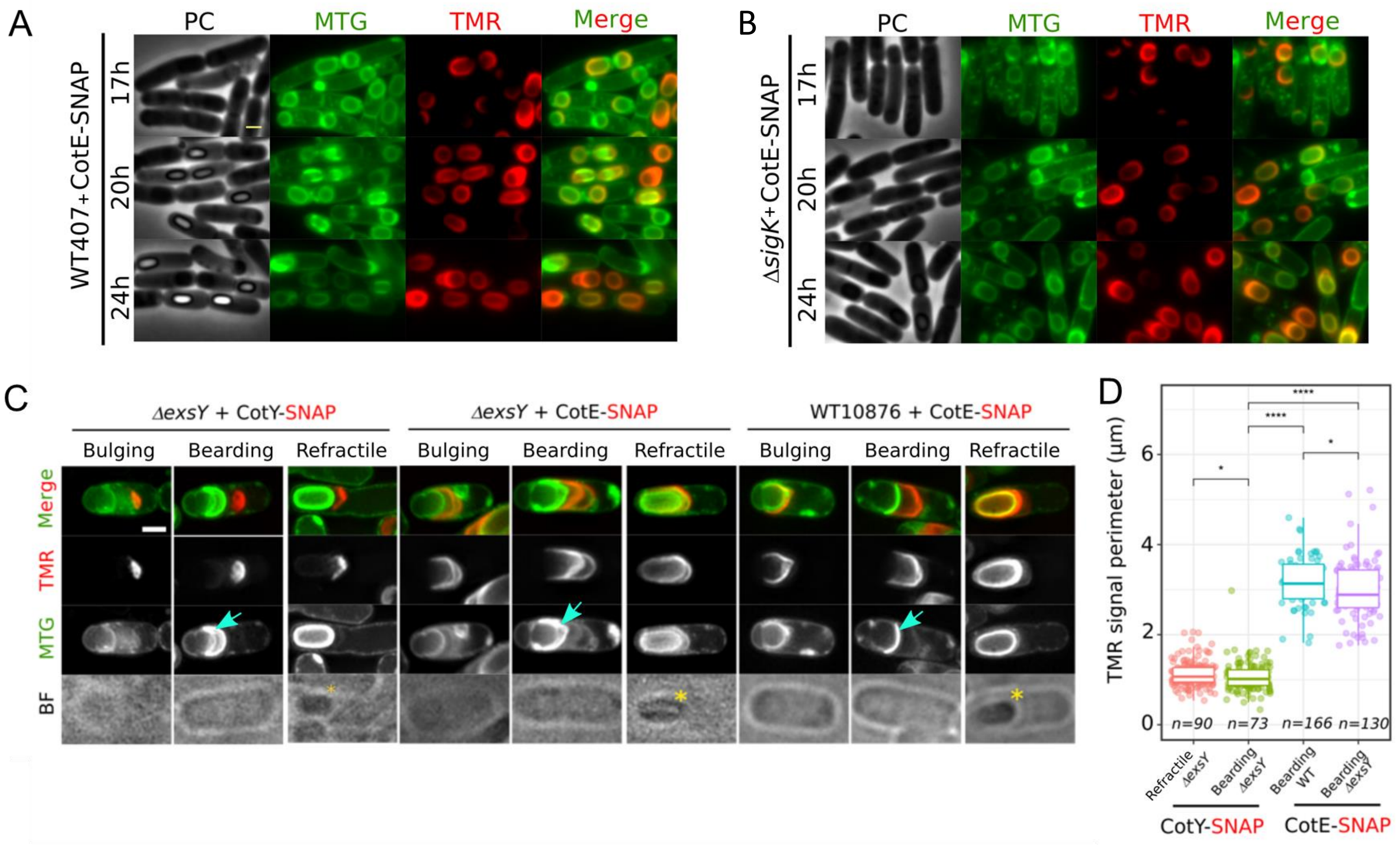

Figure S2. The σ^K–independent CotE-SNAP encasement partly occurs in engulfment-defective sporangia. WT407+CotE-SNAP (**A**), congenic Δ*sigK*+CotE-SNAP cells (**B**), sporulating at 20°C were collected at indicated times, labeled with TMR-star (red) and MTG (green) dyes and imaged by conventional fluorescence and phase contrast (PC) microscopy. A complete encasement by CotE-SNAP is observed in Δ*sigK* and WT cells. (**C-D**) Δ*exsY*+CotY-SNAP, Δ*exsY*+CotE-SNAP or WT10876+CotE-SNAP cells, sporulating at 20°C were collected at hour 48 and labeled with the TMR-star and MTG dyes and imaged by SIM and BF microscopy. In engulfed Δ*exsY* cells ("Refractile"), CotY-SNAP encasement is blocked, while encasement by CotE-SNAP is completed, similarly to the WT ("Refractile"). In "bearding" cells, encasement by CotY-SNAP is also blocked, drawing a small cap, as observed in sporangia with a refractile forespore. In contrast, CotE-SNAP assembles as a large array in both bearding Δ*exsY* and in WT bearding cells, indicating that extension of the CotE-SNAP layer, usually observed only after engulfment completion, occurs in engulfment-defective cells. MTG bright signals in bearding cells are pointed by cyan arrows and yellow asterisks indicate refractile forespore. (**D**) Boxplots showing quantification of TMR-star signal perimeter used as a proxy of SNAP fusion encasement. Bulging and bearding cells were defined based on the FS/MC MTG signal ratio calculation, see Materials and methods. Each dot represents a sporangium. Scale bars in A and C represent 1μm. The non parametric Mann-Whitney U test was used, ns, non significant; *, $p < 0.05$, ****, $p < 0.00005$, n; numbers of sporangia used for quantification.

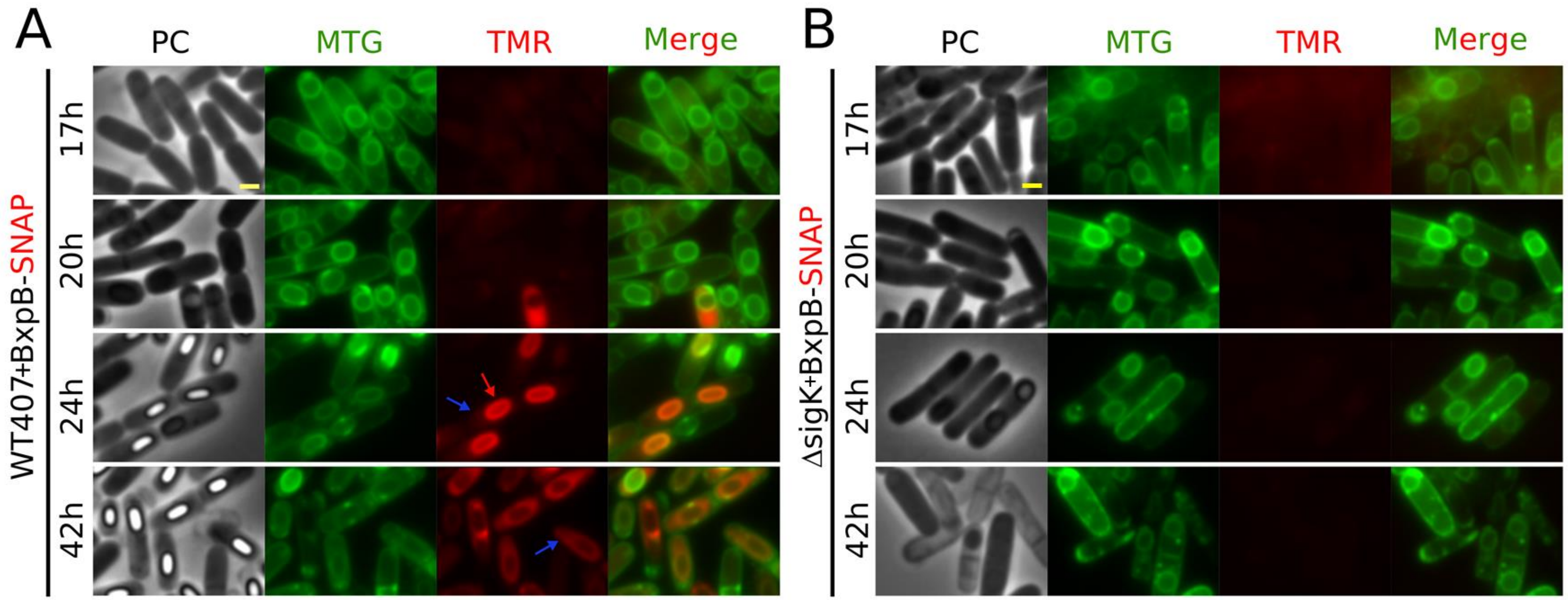

C

GTCGACAAACTAAATAATGAGTTAAGCATGGATTGGGTGGCAGAGTATTCTGCCACCCAATCCATGCTTAAAGAGTATTCTTATGTAAATTTCTTAAAATTGGGAACTTGTCTAGA
ACATAGAACCTGTCCTTTTCATTAACTGAAAGTAGAAACAGATAAAGGAGTGAAAAAC**ATG**TTCTCTTCTGATTGCGAATTTACTAAAATCGATTGCGAGGCGAAGCCAGCTAGTA
CATTACCTGCCTTCGGTTTTGCTTTCAACGCGTCTGCACCTCAGTTCGCTTCACTATTTACACCACTATTATTACCTAGCGTAAGTCCAAACCCAAATATTACTGTTCCTGTAATA
AATGATACAGTAAGTGTTGGAGATGGCATTCGAATACTACGAGCTGGTATTTATCAAATCAGTTATACATTAACGATTAGTCTTGATAACGCACCTGTAGCACCAGAAGCTGGTCG
TTTCTTCTTATCATTAGGTACACCAGCTAACATTATTCCTGGATCAGGTACAGCGGTTCGTTCTAACGTTATTGGTACTGGTGAAGTAGACGTATCCAGCGGTGTTATTCTTATTA
ACTTAAACCCTGGTGACTTAATTCAAATTGTACCAGTTGAGTTAATTGGAACTGTAGACATCCGTGCAGCAGCATTAACAGTTGCACAAATTAGCGCAGCTGCTGATAAAGATTGT
GAAATGAAGAGAACCACACTTGACAGCCCATTAGGCAAATTGGAACTTTCAGGTTGCGAACAAGGACTTCATGAAATCAAATTACTTGGTAAAGGTACTAGTGCTGCTGATGCTGT
TGAAGTGCCCGCACCAGCAGCCGTTTTGGGTGGTCCAGAGCCATTAATGCAAGCAACCGCATGGCTAAATGCGTATTTTCACCAGCCTGAGGCTATAGAAGAATTTCCGGTTCCAG
CTCTTCACCATCCTGTATTTCAACAAGAATCTTTCACCAGACAAGTTTTATGGAAATTACTAAAAGTTGTCAAATTTGGGGAAGTAATATCCTATCAACAATTAGCTGCCTTAGCG
GGAAATCCCGCAGCTACTGCAGCAGTAAAGACAGCACTTTCAGGAAACCCCGTACCGATTCTTATTCCATGTCATAGAGTCGTTTCATCCAGTGGTGCGGTCGGGGGTTATGAAGG
TGGATTAGCTGTTAAAGAATGGCTTTTGGCACATGAAGGACATCGTTTGGGGAAACCAGGACTTGGG**TAA**GGAATAAAGAATTC

Figure S3. Control of BxpB-SNAP assembly in WT407 and Δ*sigK* sporulating cells. (**A**) WT407+BxpB-SNAP or (**B**) Δ*sigK*+BxpB-SNAP cells sporulating at 20°C collected at indicated times were labeled with TMR-star (red) and MTG (green) dyes and imaged by fluorescence and PC microscopy. Red arrows point to TMR-star association with the coat and blue arrows indicate specific BxpB-SNAP-TMR-star signals. At hour 24, BxpB-SNAP-TMR-star signal is minority compared to the TMR-star signal coming from binding on coat. At hour 42, an exosporium-like localization of BxpB-SNAP-TMR-star is mostly observed in sporangia of refractile forespores. In those cells, the TMR-star signal coming from binding on coat cannot be distinguished. Scale bar in A represents 1 µm (**C**) DNA sequence of *B. cereus* ATCC 14579 *bxpB*-SNAP. The *bxpB* coding sequence (in green) and a 169bp-upstream region (in black) were fused to a linker (in red) and the *snap-tag* sequence (in blue) and cloned between the *Sal*I (in orange bold) and *Eco*RI (in purple bold) sites of pHT304-18 plasmid. Start and stop codons are in bold.
